# The Association Between Body Composition, Overall Survival, Treatment Decisions, and Patient‐Reported Outcomes in Metastatic Non‐Small‐Cell Lung Cancer

**DOI:** 10.1002/cam4.70534

**Published:** 2025-01-07

**Authors:** Adriana M. Coletta, Hyejung Lee, Sonam Puri, Sinead Culleton, Matthew F. Covington, Jeffrey T. Yap, Kelsey E. Maslana, Benjamin Haaland, Wallace Akerley

**Affiliations:** ^1^ Department of Health and Kinesiology The University of Utah Salt Lake City Utah USA; ^2^ The Huntsman Cancer Institute at the University of Utah Salt Lake City Utah USA; ^3^ Division of Biostatistics, Department of Population Health Sciences The University of Utah Salt Lake City Utah USA; ^4^ Division of Oncology, Department of Medicine The University of Utah Salt Lake City Utah USA; ^5^ Department of Radiology and Imaging Sciences The University of Utah Salt Lake City Utah USA

**Keywords:** body composition, cancer survival, treatment decisions

## Abstract

**Introduction:**

The purpose of this study was to evaluate the association between body composition, overall survival, odds of receiving treatment, and patient‐reported outcomes (PROs) in individuals living with metastatic non‐small‐cell lung cancer (mNSCLC).

**Methods:**

This retrospective analysis was conducted in newly diagnosed patients with mNSCLC who had computed‐tomography (CT) scans and completed PRO questionnaires close to metastatic diagnosis date. Cox proportional hazard models and logistic regression evaluated overall survival and odds of receiving treatment, respectively. Hazard ratios (HR) and odds ratios (OR) were evaluated as the interquartile range for body composition compartments. Multiple linear regression evaluated the association between PROs and body composition. Models were adjusted for gender, age at diagnosis, smoking history, and mutation status. The survival model also included adjustment for tumor histology.

**Results:**

Our sample (*n* = 69) included men (52%) and women (48%), with a median age of 67.4‐years, history of smoking (67%), wild‐type genotype (75.4%), and a tumor histology of adenocarcinoma (68%). Greater skeletal muscle area was associated with higher physical function scores. Larger intermuscular adipose tissue area was associated with higher mortality risk (HR 2.03, 95% CI 1.32, 3.11), lower odds of receiving treatment (OR 0.76, 95% CI 0.61, 0.93), and higher fatigue. Larger subcutaneous adipose tissue area was associated with lower mortality risk (HR 0.42, 95% CI 0.22, 0.82) and higher odds of receiving treatment (OR 1.03, 95% CI 1.01, 1.06). Larger total adipose tissue area was linked with improved survival (HR 0.59, 95% CI 0.36, 0.96).

**Conclusion:**

Findings support an association between different body composition compartments at mNSCLC diagnosis and survival, decisions to treat, and PROs. This work supports the use of data collected in routine CT scans and PROs to inform treatment decisions and supportive care options.

## Abstract 2

Findings support adiposity as a protective factor for mNSCLC survival and higher odds of receiving cancer treatment. This study supports future work aimed at utilizing multiple clinical factors (e.g., body composition and PROs), along with ECOG Performance Status, to identify a more refined method to dictate treatment decisions and prognostic information.

## Graphical abstract

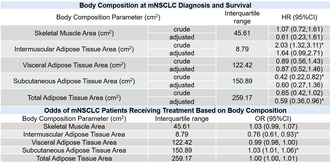

## 1 Introduction

Lung cancer with metastases is the most common cause of cancer death in the United States [1, 2]. The most common histologic subtype of lung cancer is non‐small‐cell lung cancer [2]. Mortality is due to the 55% who present with metastases and those with early‐stage or locally advanced disease who subsequently relapse. While new therapies have improved outcomes, the five‐year survival rate is still only 7% [1]. The first step to treating individuals living with metastatic non‐small‐cell lung cancer (mNSCLC) is to assess the Eastern Cooperative Oncology Group (ECOG) Performance Status (PS) scale. The ECOG PS scale is primarily defined by the oncologist's interpretation of an individuals' symptoms and time spent “out of bed” to stratify patients' ability to tolerate cancer therapy [3]. Typically, individuals with a PS of zero to two are given cancer treatment, while those with a PS equal to 3 or 4 are referred to hospice care. Often, clinical trials are more restrictive and limit eligibility to those with PS of 0 or 1 [3]. Considering the high prevalence and poor survival rate of mNSCLC, along with the strong implications PS has on cancer care, identification of objective clinical characteristics and/or patient‐centered tools that provide insight into survival rates, to complement the physician's subjective rating of PS, may yield more accurate assessment and positively impact treatment decisions.

Recent evidence suggests that body composition variables and patient‐reported outcomes (PROs) are possible independent prognostic factors of overall survival in varying cancer types and stages, including metastatic lung cancer [4, 5, 6, 7, 8, 9, 10]. Body composition is an objective clinical measure that quantifies the amount of skeletal muscle mass and fat mass that contribute to total body mass. Body composition variables can be measured by x‐ray computed tomography (CT) scans [11], scans that are performed serially as standard of care in mNSCLC. In addition, PRO measures are tools that can gather information on cancer‐related symptoms and treatment side effects (e.g., fatigue, physical function, and depression) [12]. The use of PRO measures to evaluate PROs eliminates the layer of physician interpretation. Since evidence suggests the utility of body composition and PROs as independent prognostic indicators of cancer survival [4, 5, 6, 7, 8, 9, 10], their association should be more fully explored, especially as it relates to prognostics and treatment decisions. The purpose of this investigation was to evaluate the association between body composition at mNSCLC diagnosis, overall survival, odds of receiving treatment, and PROs, in individuals living with mNSCLC.

## 2 Materials and Methods

### 2.1 Study Design and Patient Population

This retrospective analysis was carried out in a convenience sample of newly diagnosed metastatic lung cancer patients who met the following criteria: (1) a standard of care CT scan that occurred between 60 days prior to and 30 days after a metastatic lung cancer diagnosis, and (2) completed PRO questionnaires within 90 days of metastatic diagnosis date. This inclusion criteria resulted in 70 individuals eligible for the analysis. An additional individual was excluded due to insufficient CT scan quality. A total of 69 individuals were included in this analysis. This study was approved by the University of Utah Institutional Review Board (IRB #108345).

### 2.2 Patient Characteristics, CT Scans, and PROs

Gender, age at metastatic diagnosis, smoking history, mutation status, and cancer diagnosis were extracted from the medical record. Standard‐of‐care CT scans were analyzed in a single axial slice at the middle of the L3 vertebrae to segment the following tissues: skeletal muscle, intermuscular adipose tissue, visceral adipose tissue, subcutaneous adipose tissue. Cross‐sectional area (cm^2^) and radiodensity (mean Hounsfield Units [HU]) were measured for each tissue type. The CT scans were analyzed with slice‐O‐matic 5.0 rev 13 (Tomovision, Magog, CA). Predefined thresholds of HU were used to initially segment each tissue type based on the “Alberta Protocol” as follows: skeletal muscle: −29 to 150; intermuscular adipose tissue: −190 to −30, visceral adipose tissue: −150 to −50; and subcutaneous adipose tissue: −190 to −30. A radiologist then reviewed the results of each segmentation and manually refined the segmentations as needed. Additionally, the following PROMIS (Patient‐Reported Outcomes Measurement Information System) questionnaires as part of a quality improvement project were completed by patients at home prior to attending their standard of care (SOC) clinic visit or upon check‐in at their SOC visit: Physical Function, Depression, Anxiety, Pain, and Fatigue. All patients received an email prior to their SOC clinic visit requesting they complete the PROMIS questionnaires. For individuals who did not complete the questionnaires in advance, an iPad was provided in the clinic after check‐in for questionnaire completion.

### 2.3 Statistical Analysis

Descriptive statistics were used to evaluate patient characteristics and are presented as either frequencies, mean and standard deviation, median and interquartile range, or range. Cox proportional hazard models were used to evaluate overall survival (OS). Adjusted survival models included gender, age at metastasic diagnosis, smoking history, mutation status, and tumor histology. Some commonly identified risk factors of OS in mNSCLC patients, such as treatment types, were not adjusted in the model because they are postbaseline (i.e., postdiagnosis) variables. The main exposure variables in this analysis are body composition compartments measured at diagnosis, and cancer treatment is given after diagnosis. Body composition may have affected the provider's decision for treatment, which in turn affects overall survival. Therefore, adjusting for treatment information can either falsely induce/amplify or falsely reduce/remove the observed association between body composition compartments and survival. Thus, no postbaseline variables are adjusted in the survival model including the treatment types. Additionally, the range of body composition compartment areas are vastly different from one another. For example, in our dataset, we observed that most of the intermuscular adipose tissue areas ranged from 3.2 to 12.0 cm^2^, whereas most of the skeletal muscle areas ranged between 94.7 and 140.3 cm^2^. This can be interpreted such that a 1‐unit difference in intermuscular adipose tissue area has a more meaningful difference than a 1‐unit difference in skeletal muscle area. To create a uniform interpretation of a meaningful difference across body composition compartments, we evaluated hazard ratios as the difference between the 75th percentile (i.e., moderately high value) and 25th percentile (i.e., moderately low value) of the distribution (interquartile range) for each compartment (e.g., the risk of the 75th percentile compared with the 25th percentile [reference group]). In addition, logistic regression was used to evaluate the odds of receiving treatment for the difference between the 75th and 25th percentiles for each body composition compartment.

Models for skeletal muscle area, intermuscular adipose tissue area, visceral adipose tissue area, and subcutaneous adipose tissue area considered all body composition variables at the same time as opposed to individually. We calculated total adipose tissue area (cm^2^) as the addition of visceral adipose tissue area and subcutaneous adipose tissue area. Further, multiple linear regression was used to evaluate the association between PROs and body composition parameters. Only patients who completed the survey within 90 days following metastatic diagnosis were included in this analysis. Models were adjusted for gender, age at metastatic diagnosis, smoking history, and mutation status. For all analyses, statistical significance was defined to be any *p* values ≤ 0.05. All data were analyzed utilizing the R statistical software (version 4.4.0).

## 3 Results

### 3.1 Patient Characteristics

Patient characteristics are presented in Table 1. Our sample included men (52%) and women (48%), with a median age at metastatic diagnosis of 67.4 years. Most patients had a history of smoking (67%), a lung cancer mutation status that was nonactionable with targeted therapy (wild‐type, 75%), and a tumor histology of adenocarcinoma (68%). The median and interquartile range for skeletal muscle, visceral fat, subcutaneous fat, and intermuscular fat areas were 116.9 cm^2^ (94.7, 140.3), 116.9 cm^2^ (51.3, 173.7), 150.7 cm^2^ (88.2, 239.1), and 6.2 cm^2^ (3.2, 12.0), respectively. PROs within 90 days of diagnosis revealed the following average scores (± standard deviation): physical function 37.3 ± 9.0, depression 54.5 ± 8.4, anxiety 60.2 ± 8.9, pain 57.9 ± 9.4, and fatigue 60.6 ± 9.5. A median score is anticipated to be 50 in the general population and higher scores mean greater symptom load, except for physical function where higher scores are reflective of greater ability.

**TABLE 1:** Patient characteristics.

Variable	Summary
Gender *n* (%)	
Female	33 (48%)
Male	36 (52%)
Age at metastasis diagnosis	
Mean (SD)	66.1 (11.4)
Median (IQR)	67.4 (57.7, 73.6)
Range	38.6, 89.8
Smoking history *n* (%)	
History of smoking	46 (67%)
No history of smoking	23 (33%)
Mutation status *n* (%)	
Mutation	17 (25%)
Wild‐type	52 (75%)
BMI (kg/m^2^; *n* = 68)	
Mean (SD)	25.3 (4.7)
Median (IQR)	24.8 (21.1, 29.1)
Range	16.7 (36.7)
BMI (categorical; *n* = 68)^a^	
Underweight	3 (4.4%)
Normal weight	32 (47.1%)
Overweight	22 (32.4%)
Obesity Class I	10 (14.7%)
Obesity Class II	1 (1.5%)
Tumor histology *N* (%)	
Adenocarcinoma	47 (68%)
Non‐squamous NSCLC	11 (16%)
Squamous cell carcinoma	11 (16%)
Treatment history *n* (%)	
Chemotherapy	37 (54%)
Chemotherapy + Immunotherapy	5 (7%)
Investigational (immunotherapy)	1 (1%)
Targeted therapy	17 (25%)
Not treated	9 (13%)
Skeletal muscle area (cm^2^)	
Mean (SD)	121.4 (34.3)
Median (IQR)	116.9 (94.7, 140.3)
Range	74.8‐199.5
Intermuscular adipose tissue area (cm^2^)	
Mean (SD)	8.2 (6.7)
Median (IQR)	6.2 (3.2, 12.0)
Range	0.7‐35.5
Visceral adipose tissue area (cm^2^)	
Mean (SD)	128.5 (93.8)
Median (IQR)	116.9 (51.3, 173.7)
Range	11.8‐417.6
Subcutaneous adipose tissue area (cm^2^)	
Mean (SD)	171.6 (99.2)
Median (IQR)	150.7 (88.2, 239.1)
Range	7.2‐446.7
Patient reported outcomes	
Physical function (*n* = 68)	
Mean (SD)	37.3 (9.0)
Median (IQR)	38.1 (31.0, 45.2)
Range	14.6‐50.2
Depression (*n* = 67)	
Mean (SD)	54.5 (8.4)
Median (IQR)	55.0 (49.9, 60.2)
Range	36.8‐76.4
Anxiety (*n* = 68)	
Mean (SD)	60.2 (8.9)
Median (IQR)	61.0 (52.5, 66.9)
Range	33.7‐79.0
Pain (*n* = 68)	
Mean (SD)	57.9 (9.4)
Median (IQR)	58.1 (50.8, 65.5)
Range	37.9‐76.4
Fatigue (*n* = 68)	
Mean (SD)	60.6 (9.5)
Median (IQR)	60.7 (54.9, 66.0)
Range	40.4‐84.8

*Note: N* = 69 for descriptive variables. NSCLC = non‐small cell lung cancer. Missing values: BMI *n* = 1, BMI (categorical) *n* = 1, physical function *n* = 1, depression *n* = 2, anxiety *n* = 1, pain *n* = 1, fatigue *n* = 1.

^a^ BMI categories: underweight < 18.5 kg/m^2^, normal weight = 18.5–24.9 kg/m^2^, overweight = 25–29.9 kg/m^2^, obesity Class I = 30–34.9 kg/m^2^, obesity Class II = 35–39.9 kg/m^2^.

### 3.2 The Association Between Body Composition, Overall Survival, and Odds of Receiving Treatment

The association between body composition and overall survival is presented in Table 2. In the crude models, significant findings were observed for L3 intermuscular adipose tissue area such that compared to individuals with moderately small intermuscular adipose tissue area, individuals with moderately large intermuscular adipose tissue area (greater than or equal to 8.79 cm^2^) experienced a twofold increase in mortality risk (HR 2.03, 95% CI 1.32, 3.11). In contrast, for subcutaneous adipose tissue area, compared to individuals with moderately small subcutaneous adipose tissue area, individuals with moderately large subcutaneous adipose tissue area (greater than or equal to 150.89 cm^2^) experienced a 58% reduction in mortality risk (HR 0.42, 95% CI 0.22, 0.82). A significant association was lost for both compartments in adjusted models. Adjusted models revealed a significant association between total adipose tissue area and improved survival (HR 0.59, 95% CI 0.36, 0.96). No other statistically significant associations were observed.

**TABLE 2:** Body composition and overall survival.

Body composition parameter (cm^2^)	Interquartile range	HR (95% CI)
Skeletal muscle area (cm^2^)		
Crude	45.61	1.07 (0.72,1.61)
Adjusted		0.61 (0.23, 1.61)
Intermuscular adipose tissue area (cm^2^)		
Crude	8.79	2.03 (1.32, 3.11)*
Adjusted		1.64 (0.99, 2.71)
Visceral adipose tissue area (cm^2^)		
Crude	122.42	0.89 (0.56, 1.43)
Adjusted		0.87 (0.52, 1.46)
Subcutaneous adipose tissue area (cm^2^)		
Crude	150.89	0.42 (0.22, 0.82)*
Adjusted		0.60 (0.27, 1.36)
Total adipose tissue area (cm^2^)		
Crude	259.17	0.65 (0.42, 1.02)
Adjusted		0.59 (0.36, 0.96)*

*Note:* For each body composition compartment, two models, crude and adjusted, were fit. All adjusted models considered gender, age at metastasis diagnosis, history of smoking, mutation status, and tumor histology. Models for skeletal muscle area, intermuscular adipose tissue area, visceral adipose tissue area, and subcutaneous adipose tissue area also considers all body composition variables at the same time as opposed to individually. The hazard ratio (HR) is provided as the risk of 3rd quartile compared to the 1st quartile (reference group) (e.g., interquartile range). Total Adipose Tissue Area (cm^2^) was calculated as the addition of Visceral Adipose Tissue Area and Subcutaneous Adipose Tissue Area.

* Statistical significance (*p* < 0.05).

When evaluating the radiodensity (HU) of body composition parameters, a significant association was observed in the crude model for skeletal muscle radiodensity, where individuals with moderately high skeletal muscle radiodensity (i.e., difference in 13.19 HU) experienced a 39% reduction in mortality risk (HR 0.61, 95% CI 0.38, 0.98). However, this effect was lost in the adjusted model. No other statistically significant associations were observed (Table S1).

The association between body composition and odds of receiving treatment is outlined in Table 3. Individuals with moderately large intermuscular adipose tissue area was associated with lower odds of receiving treatment (OR 0.76, 95% CI 0.61, 0.93). Additionally, a moderately large subcutaneous adipose tissue area was significantly associated with higher odds of receiving treatment (OR 1.03, 95% CI 1.01, 1.06). Further, when evaluating radiodensity, both skeletal muscle and intermuscular adipose tissue were positively associated with increased odds of receiving treatment. No other statistically significant associations were observed (Table S2).

**TABLE 3:** Odds of receiving treatment based on body composition.

Body composition parameter (cm^2^)	Interquartile range	OR (95% CI)
Skeletal muscle area	45.61	1.03 (0.99, 1.07)
Intermuscular adipose tissue area	8.79	0.76 (0.61, 0.93)*
Visceral adipose tissue area	122.42	0.99 (0.98, 1.00)
Subcutaneous adipose tissue area	150.89	1.03 (1.01, 1.06)*
Total adipose tissue area	259.17	1.00 (1.00, 1.01)

*Note:* Models for skeletal muscle area, intermuscular adipose tissue area, visceral adipose tissue area, and subcutaneous adipose tissue area also considers all body composition variables at the same time as opposed to individually. The odds ratio (OR) is provided as the odds of 3rd quartile compared to the 1st quartile (reference group). Total adipose tissue area (cm^2^) was calculated as the addition of visceral adipose tissue area and subcutaneous adipose tissue area.

* Statistical significance (*p* < 0.05).

### 3.3 The Association Between Body Composition and PROs

The association between body composition and PROs is presented in Table 4. Skeletal muscle area was found to be positively associated with physical function in the crude and adjusted models (crude: 0.07 cm^2^, 95% CI 0.01, 0.14; adjusted: 0.10 cm^2^, 95% CI 0.01, 0.20). Intermuscular adipose tissue area was found to be positively associated with fatigue in both crude and adjusted models (crude: 0.37 cm^2^, 95% CI 0.04, 0.70; adjusted: 0.36 cm^2^, 95% CI 0.02, 0.69). No other statistically significant associations were observed. When considering radiodensity, an inverse association between skeletal muscle and depression was observed in the crude model, and skeletal muscle and fatigue in both the crude and adjusted models. Additionally, visceral adipose tissue and subcutaneous adipose tissue were positively associated with pain in both crude and adjusted models (Table S3).

**TABLE 4:** Patient‐reported outcomes (PRO) based on body composition.

PRO	Body composition parameter (cm^2^)	Mean (95% CI)
Physical function	Skeletal muscle area	
	Crude	0.07 (0.01, 0.14)*
	Adjusted	0.10 (0.01, 0.20)*
	Intermuscular adipose tissue area	
	Crude	−0.20 (−0.56, 0.17)
	Adjusted	−0.09 (−0.46, 0.28)
	Visceral adipose tissue area	
	Crude	0.01 (−0.01, 0.03)
	Adjusted	0.01 (−0.01, 0.04)
	Subcutaneous adipose tissue area	
	Crude	−0.00 (−0.02, 0.02)
	Adjusted	−0.01 (−0.03, 0.01)
Depression	Skeletal muscle area	
	Crude	−0.02 (−0.08, 0.04)
	Adjusted	0.02 (−0.08, 0.12)
	Intermuscular adipose tissue area	
	Crude	0.22 (−0.08, 0.53)
	Adjusted	0.16 (−0.16, 0.48)
	Visceral adipose tissue area	
	Crude	0.02 (−0.01, 0.04)
	Adjusted	0.02 (−0.01, 0.04)
	Subcutaneous adipose tissue area	
	Crude	0.01 (−0.01, 0.03)
	Adjusted	0.02 (−0.01, 0.04)
Anxiety	Skeletal muscle area	
	Crude	−0.04 (−0.10, 0.02)
	Adjusted	−0.06 (−0.16, 0.04)
	Intermuscular adipose tissue area	
	Crude	0.07 (−0.25, 0.39)
	Adjusted	0.02 (−0.30, 0.35)
	Visceral adipose tissue area	
	Crude	−0.00 (−0.02, 0.02)
	Adjusted	−0.00 (−0.02, 0.02)
	Subcutaneous adipose tissue area	
	Crude	−0.00 (−0.02, 0.02)
	Adjusted	0.01 (−0.02, 0.03)
Pain	Skeletal muscle area	
	Crude	−0.03 (−0.10, 0.03)
	Adjusted	−0.11 (−0.22, 0.00)
	Intermuscular adipose tissue area	
	Crude	0.02 (−0.32, 0.36)
	Adjusted	0.04 (−0.32, 0.39)
	Visceral adipose tissue area	
	Crude	−0.02 (−0.05, 0.00)
	Adjusted	−0.03 (−0.05, 0.00)
	Subcutaneous adipose tissue area	
	Crude	−0.01 (−0.04, 0.01)
	Adjusted	−0.01 (−0.03, 0.02)
Fatigue	Skeletal muscle area	
	Crude	−0.01 (−0.08, 0.05)
	Adjusted	−0.05 (−0.16, 0.05)
	Intermuscular adipose tissue area	
	Crude	0.37 (0.04, 0.70)*
	Adjusted	0.36 (0.02, 0.69)*
	Visceral adipose tissue area	
	Crude	0.00 (−0.02, 0.03)
	Adjusted	−0.00 (−0.03, 0.02)
	Subcutaneous adipose tissue area	
	Crude	0.01 (−0.01, 0.03)
	Adjusted	0.02 (−0.00, 0.05)

*Note:* For each PRO, two models, crude and adjusted, were fit. All adjusted models considered gender, age at metastasis diagnosis, history of smoking, and mutation status. Only patients who completed the survey within 90 days following metastatic diagnosis were included in this analysis.

* Statistical significance (*p* < 0.05).

## 4 Discussion

We aimed to investigate the association between body composition, overall survival, the likelihood of treatment, and PROs in individuals with mNSCLC. We observed a significant positive association between subcutaneous adipose tissue area, total adipose tissue area, and survival. However, we also observed that individuals with greater subcutaneous adipose tissue area were more likely to receive treatment, suggesting at least in the mNSCLC setting that perhaps individuals with a phenotype suggesting higher body fat experience improved survival because they are more likely to receive treatment. In a recent pooled analysis of 22 clinical trials among men and women living with advanced NSCLC who received treatment, those with a BMI ≥ 25 kg/m^2^ had greater overall survival compared with those with a BMI < 25 kg/m^2^ [13]. Yet in this trial, individuals receiving carboplatin/paclitaxel had greater overall survival compared with those who received Cisplatin + Vinorelbine therapy. Taken together, further evaluation is needed to decide whether the better outcome is due to greater physiologic reserve, the cancer's effect on adipose tissue, and consideration of treatment type if treatment was received.

There are many factors that come into consideration when making cancer treatment decisions. The ability to accurately identify patients who may or may not benefit from targeted or nontargeted therapies with palliation, tolerate therapy, and experience improved survival is of utmost importance. One of the primary contributors to making this decision is ECOG performance status (PS), which reflects a physician's interpretation of an individual's functional status and independence. Body composition, as measured by routine CT scans, may serve as an objective measure to complement ECOG PS and inform treatment decisions. In mNSCLC, chemotherapy and targeted therapy are typically accompanied by significant losses in skeletal muscle mass and fat mass that may be reversible if therapies have been effective [9, 14]. The observed loss in skeletal muscle mass is coupled with a loss in fat mass and associated with poor overall survival, independent of PS and other important prognostic clinical factors such as gender and age [9]. We found that individuals with large subcutaneous adipose tissue area (≥ 150.89 cm^2^) have higher odds to receive treatment, suggesting that individuals with a frail or cachectic body composition may have negatively influenced the decision to treat, even though body composition is not an element considered by PS. Additionally, we observed that individuals with higher intermuscular adipose tissue, an indication of impaired muscular health and metabolic dysfunction [15], were 24% more likely not to receive treatment (Table 3). Objective assessment of body composition may serve as an important complement to ECOG PS to help inform treatment decisions.

Additionally, using PROs, in conjunction with CT‐measured body composition and physician‐rated performance status, can foster a patient‐centered approach of shared decision‐making for treatment decisions. Work from our institution and others have previously demonstrated that the PRO domain of physical function is the most predictive of overall survival [4, 5, 7, 10]. Considering this information, we expected to observe an association between physical function and body composition, notably a higher physical function score, or better physical function, would be associated with greater skeletal muscle mass. Findings from our crude and adjusted models supported this hypothesis, where in our adjusted model for example, for every one area (cm^2^) increase in skeletal muscle, the physical function score increased by 0.1 points.

We noted a positive affiliation between pain scores and both visceral and subcutaneous adipose tissue radiodensity (HU), but not area of these compartments. Higher radiodensity of adipose tissue compartments is indicative of lower lipid content, higher vascularity, and greater inflammation [16, 17, 18]. Since lower lipid content may be associated with a frail or cachectic phenotype, this may explain a lower pain tolerance in these individuals. Additionally, pain scores were inversely associated with skeletal muscle area in adjusted models. We were interested to observe that the CT scan might be able to infer general symptoms of pain or fatigue.

In contrast to the literature, in our models for visceral adipose tissue and subcutaneous adipose tissue radiodensity, we did not observe a significant association between higher radiodensity of these fat compartments (i.e., lower lipid content) and overall survival. These findings are inconsistent with the literature in other cancer types (e.g., nonmetastatic colorectal cancer [17], nonmetastatic esophageal cancer [19], and liver cancer [20]). Taken together, visceral and subcutaneous adipose tissue radiodensity may have some prognostic value, but more work must be done in this area.

We are the first group, to our knowledge, to evaluate radiodensity of body composition parameters as they relate to PROs, and within the mNSCLC setting. We consider this a strength of our work. The addition of PROs and analysis of radiodensity may help provide further insight into mNSCLC survival, treatment decisions, and the patient perspective. Additionally, evaluation of hazard ratios as the difference between 75th and 25th percentiles resulted in a uniform interpretation of a meaningful difference across body composition compartments, making our work more comprehensible. Yet, our work is not without limitations. A larger sample size is needed to increase the generalizability of our findings. Additionally, our analysis consisted of a convenience sample from one provider's clinic, which may bias our results. Further, considering there are other variables that may impact overall survival, such as concomitant therapy, it is important to note that this work does not establish causality regarding body composition at mNSCLC diagnosis as a prognostic variable for survival. Future work is needed with a larger sample size, a more heterogeneous sample of mNSCLC patients, evaluation of change in body composition throughout treatment, and consideration of other variables that may impact overall survival.

## 5 Conclusion

Together, our findings support adiposity as a protective factor in the context of mNSCLC survival. Additionally, our findings may contribute to future work aimed at utilizing multiple clinical factors (e.g., body composition and PROs), along with ECOG PS, and other variables that may impact survival, to identify a more refined method to dictate treatment decisions and prognostic information.

## Author Contributions

**Adriana M. Coletta:** conceptualization (supporting), investigation (equal), project administration (lead), writing – original draft (lead), writing – review and editing (lead). **Wallace Akerley:** conceptualization (lead), data curation (lead), funding acquisition (lead), investigation (equal), project administration (supporting), writing – original draft (supporting), writing – review and editing (equal). **Benjamin Haaland:** conceptualization (supporting), formal analysis (supporting), investigation (equal), methodology (equal), writing – review and editing (supporting). **Kelsey E. Maslana:** writing – review and editing (equal). **Sonam Puri:** investigation (equal), writing – review and editing (equal). **Hyejung Lee:** conceptualization (supporting), formal analysis (lead), investigation (equal), methodology (lead), writing – review and editing (equal). **Sinead Culleton:** data curation (equal), investigation (equal), writing – review and editing (equal). **Jeffrey T. Yap:** conceptualization (supporting), data curation (equal), investigation (equal), writing – review and editing (equal). **Matthew F. Covington:** data curation (equal), investigation (equal), writing – review and editing (equal).

## Ethics Statement

This study was performed in line with the principles of the Declaration of Helsinki. Approval was granted by the Ethics Committee of the University of Utah (IRB #108345). The Ethics Committee of the University of Utah waived informed consent procedures for this protocol since the data utilized were from standard‐of‐care procedures.

## Conflicts of Interest

The authors declare no conflicts of interest.

## Precis

Findings support adiposity as a protective factor for mNSCLC survival and higher odds of receiving cancer treatment. This study supports future work aimed at utilizing multiple clinical factors (e.g., body composition and PROs), along with ECOG Performance Status, to identify a more refined method to dictate treatment decisions and prognostic information.

## Supporting information


Table S1.

Table S2.

Table S3.

## Data Availability

De‐identified data are available upon request.
