# Supplementary material for: The Association Between Body Composition, Overall Survival, Treatment Decisions, and Patient‐Reported Outcomes in Metastatic Non‐Small‐Cell Lung Cancer
Source: Cancer Med. 2025 Jan 7;14(1):e70534. doi: 10.1002/cam4.70534 (PMC11705441; doi:10.1002/cam4.70534)
Supplement: Supplementary file 1 — Table S1. [file CAM4-14-e70534-s003.docx]

**Supplementary Table 1: Body Composition in HU and Overall Survival**

| **Body Composition Parameter (Mean HU)** | | **Interquartile range** | **HR (95%CI)** |
| --- | --- | --- | --- |
| Skeletal Muscle Radiodensity | crude | 13.19 | 0.61 (0.38,0.98)* |
|  | adjusted |  | 0.82 (0.48,1.40) |
| Intermuscular Adipose Tissue Radiodensity | crude | 6.89 | 0.88 (0.64,1.22) |
|  | adjusted |  | 1.11 (0.77,1.58) |
| Visceral Adipose Tissue Radiodensity | crude | 12.14 | 2.10 (0.89,4.98) |
|  | adjusted |  | 2.12 (0.89,5.03) |
| Subcutaneous Adipose Tissue Radiodensity | crude | 13.81 | 1.18 (0.56,2.50) |
|  | adjusted |  | 0.98 (0.42,2.29) |

For each body composition parameter, two models, crude and adjusted, were fit. All adjusted models considered gender, age at metastasis diagnosis, history of smoking, mutation status, and tumor histology. Models for skeletal muscle area, intermuscular adipose tissue area, visceral adipose tissue area, and subcutaneous adipose tissue area also considers all body composition variables at the same time as opposed to individually. The hazard ratio (HR) is provided as the risk of 3^rd^ quartile compared to the 1^st^ quartile (reference group)(e.g., interquartile range). *Statistical Significance (p<0.05)
