# Supplementary material for: The Association Between Body Composition, Overall Survival, Treatment Decisions, and Patient‐Reported Outcomes in Metastatic Non‐Small‐Cell Lung Cancer
Source: Cancer Med. 2025 Jan 7;14(1):e70534. doi: 10.1002/cam4.70534 (PMC11705441; doi:10.1002/cam4.70534)
Supplement: Supplementary file 2 — Table S2. [file CAM4-14-e70534-s001.docx]

**Supplementary Table 2: Odds of Receiving Treatment Based on Body Composition**

| **Body Composition Parameter (Mean HU)** | **Interquartile range** | **OR (95%CI)** |
| --- | --- | --- |
| Skeletal Muscle Radiodensity | 13.19 | 1.20 (1.03, 1.40)* |
| Intermuscular Adipose Tissue Radiodensity | 6.89 | 1.20 (1.02,1.40)* |
| Visceral Adipose Tissue Radiodensity | 12.14 | 0.94 (0.79,1.11) |
| Subcutaneous Adipose Tissue Radiodensity | 13.81 | 0.91 (0.80,1.05) |

Models for skeletal muscle area, intermuscular adipose tissue area, visceral adipose tissue area, and subcutaneous adipose tissue area also considers all body composition variables at the same time as opposed to individually. The odds ratio (OR) is provided as the risk of 3^rd^ quartile compared to the 1^st^ quartile (reference group). Total Adipose Tissue Area (cm^2^) was calculated as the addition of Visceral Adipose Tissue Area and Subcutaneous Adipose Tissue Area. *Statistical Significance (p<0.05)
