# Supplementary material for: The Association Between Body Composition, Overall Survival, Treatment Decisions, and Patient‐Reported Outcomes in Metastatic Non‐Small‐Cell Lung Cancer
Source: Cancer Med. 2025 Jan 7;14(1):e70534. doi: 10.1002/cam4.70534 (PMC11705441; doi:10.1002/cam4.70534)
Supplement: Supplementary file 3 — Table S3. [file CAM4-14-e70534-s002.docx]

**Supplementary Table 3: Patient Reported Outcomes (PRO) by Body Composition in HU**

| **PRO** | **Body Composition Parameter (Mean HU)** | | **Mean (95%CI)** |
| --- | --- | --- | --- |
| Physical Function | Skeletal Muscle Radiodensity | crude | 0.20 (-0.02,0.43) |
|  |  | adjusted | 0.10 (-0.13,0.34) |
|  | Intermuscular Adipose Tissue Radiodensity | crude | 0.08 (-0.25,0.40) |
|  |  | adjusted | -0.08 (-0.42,0.25) |
|  | Visceral Adipose Tissue Radiodensity | crude | -0.24 (-0.47,0.00) |
|  |  | adjusted | -0.19 (-0.41,0.03) |
|  | Subcutaneous Adipose Tissue Radiodensity | crude | -0.18 (-0.36,0.01) |
|  |  | adjusted | -0.16 (-0.36,0.04) |
| Depression | Skeletal Muscle Radiodensity | crude | -0.27 (-0.47,-0.06)* |
|  |  | adjusted | -0.21 (-0.44,0.02) |
|  | Intermuscular Adipose Tissue Radiodensity | crude | -0.05 (-0.35,0.26) |
|  |  | adjusted | 0.07 (-0.27,0.41) |
|  | Visceral Adipose Tissue Radiodensity | crude | -0.08 (-0.31,0.15) |
|  |  | adjusted | -0.11 (-0.34,0.12) |
|  | Subcutaneous Adipose Tissue Radiodensity | crude | 0.02 (-0.17,0.20) |
|  |  | adjusted | -0.01 (-0.21,0.20) |
| Anxiety | Skeletal Muscle Radiodensity | crude | -0.12 (-0.34,0.11) |
|  |  | adjusted | -0.07 (-0.30,0.17) |
|  | Intermuscular Adipose Tissue Radiodensity | crude | -0.05 (-0.38,0.27) |
|  |  | adjusted | 0.00 (-0.34,0.35) |
|  | Visceral Adipose Tissue Radiodensity | crude | 0.00 (-0.24,0.23) |
|  |  | adjusted | -0.06 (-0.30,0.17) |
|  | Subcutaneous Adipose Tissue Radiodensity | crude | 0.02 (-0.17,0.21) |
|  |  | adjusted | -0.03 (-0.24,0.17) |
| Pain | Skeletal Muscle Radiodensity | crude | -0.06 (-0.30,0.18) |
|  |  | adjusted | -0.06 (-0.32,0.20) |
|  | Intermuscular Adipose Tissue Radiodensity | crude | 0.30 (-0.04,0.63) |
|  |  | adjusted | 0.34 (-0.03,0.71) |
|  | Visceral Adipose Tissue Radiodensity | crude | 0.33 (0.10,0.57)* |
|  |  | adjusted | 0.30 (0.06,0.55)* |
|  | Subcutaneous Adipose Tissue Radiodensity | crude | 0.24 (0.05,0.43)* |
|  |  | adjusted | 0.23 (0.01,0.45)* |
| Fatigue | Skeletal Muscle Radiodensity | crude | -0.31 (-0.53,-0.08)* |
|  |  | adjusted | -0.28 (-0.52,-0.04)* |
|  | Intermuscular Adipose Tissue Radiodensity | crude | 0.09 (-0.25,0.43) |
|  |  | adjusted | 0.14 (-0.23,0.51) |
|  | Visceral Adipose Tissue Radiodensity | crude | 0.08 (-0.17,0.34) |
|  |  | adjusted | -0.01 (-0.26,0.24) |
|  | Subcutaneous Adipose Tissue Radiodensity | crude | 0.08 (-0.12,0.28) |
|  |  | adjusted | -0.04 (-0.26,0.18) |

For each PRO, two models, crude and adjusted, were fit. All adjusted models considered gender, age at metastasis diagnosis, history of smoking, and mutation status. Only patients who completed the survey within 90 days following metastatic diagnosis were included in this analysis. *Statistical Significance (p < 0.05)
